# Notes and News

**Published:** 1899-03-25

**Authors:** 


### Notes and News.

(Collected and Communicated.)

HOSPITAL MEETINGS.

The Royal Eye Hospital, St. George's Circus, Southwark, S.E.—Annual General Meeting of Governors at four p.m., on Wednesday, March 29th, at the Hospital.

Samaritan Free Hospital for Women and Children, Marylebone Road.—Annual Meeting at five p.m., on Tuesday, April 18th.

Chelsea Hospital for Women, Fulham Road.—Annual Meeting at three p.m., on Thursday, May 11th.

London Homœopathic Hospital, Great Ormond Street, Bloomsbury.—Annual General Meeting at four on Friday, March 24th, in the Board Room of the Hospital.

The Right Rev. the Lord Bishop of Rochester, at a short service held in St. Thomas's Hospital Chapel on Tuesday, unveiled the memorial presented to the chapel by Mr. Lewis Doulton, in memory of his late father.

Referring to an article on "Milk Laboratories," which appeared in The Hospital for March 11th, the Aylesbury Dairy Company draw our attention to the fact that the system of modifying milk according to physicians' prescriptions, on the lines there indicated, has been in operation at their laboratories for some time.

A society is being formed, under the title of the Cancer Society, with the object of combating this terrible disease. The objects are: (1) The improvement of technical medical education; (2) popular instruction in elementary health laws bearing upon the prevention, amelioration, or cure of cancerous diseases; (3) the institution of prizes for original essays or investigations; (4) the delivery of lectures by the most eminent scientists; (5) the foundation of a special laboratory for cancer research; (6) the utilisation of existing special hospitals for teaching purposes; (7) the promotion of Parliamentary inquiry into the causes of the mortality, &c.; (8) collection and publication of reliable statistics; and (9) the establishment of a cancer home for persons of limited means.

St. Thomas's Hospital has received £1,000 anonymously to endow a bed to the memory of Thomas Hughes, Q.C.

Through the heroic conduct of Dr. Neil McPhatter, of Edinburgh, a paralysed lady, sister of the Governor of Georgia, was rescued from the fire at the Windsor Hotel, New York. Dr. McPhatter escaped himself, but not without injury, as in descending from the hotel his leg was broken.

The Marylebone Board of Guardians have unanimously adopted a report from their Schools Visiting Committee recommending that, subject to the approval of the Local Government Board, an assistant medical officer for dental purposes be appointed, whose business it would be to go down weekly and inspect the children's teeth. It was stated that statistics proved that as many as 85 per cent. of the children's teeth required attention.

Medical men in the Isle of Man are exempt from the operation of the English Medical Acts. In response to recent agitation in the island on this matter a Bill is about to be introduced into the Manx Legislative Council providing that "only persons registered under the Imperial Medical Acts can sue for or recover fees for medical attendance, or be appointed to public medical positions." The Bill also provides that persons passing unlawfully as registered practitioners shall be liable to a penalty not exceeding £20.

The Willesden Cottage Hospital is undergoing considerable alteration and enlargement, thanks in great measure to the generosity of that universal benefactor, Mr. Passmore Edwards, after whom the hospital is henceforth to be named. A café chantant in special aid of the children's ward will be given at the Portman Rooms, Baker Street, on Saturday, April 15th, from three to seven p.m., which it is hoped will go far towards securing the further funds required. The entertainment is being organised under the immediate patronage of the Countess of Grosvenor, the Countess of Cavan, Lady Ebury, Sir John and Lady Hutton, Messrs. W. Ambrose, I. Cox, and J. Passmore Edwards.

Great efforts are being made to raise a sum of £70,000 to complete the Clarence Memorial Wing of St. Mary's Hospital, of which the foundation-stone was laid by the Prince of Wales in 1892. A letter from Sir William Broadbent, Lord Rothschild, and the chairman and treasurers of the hospital points out that the new buildings are not being constructed in order to extend the sphere of the hospital, but to provide space in which the present work may be properly performed. The appeal is especially made to the richer residents of Kensington and Marylebone, which districts are largely served by St. Mary's, but which, in comparison with Paddington, do little to maintain it. The work of raising funds, now begun, will continue till the end of May, when the Duchess of Fife has promised her aid and will open a bazaar at the Portman Rooms. Contributions may be sent to any of the signatories whose names are given above, or to the secretary, Mr. Thomas Ryan, at the hospital.

